# Butyrate reduces cellular magnesium absorption independently of metabolic regulation in Caco-2 human colon cells

**DOI:** 10.1038/s41598-022-21683-6

**Published:** 2022-11-03

**Authors:** Lisanne M. M. Gommers, Pieter A. Leermakers, Jenny van der Wijst, Sara R. Roig, Anastasia Adella, Melissa A. E. van de Wal, René J. M. Bindels, Jeroen H. F. de Baaij, Joost G. J. Hoenderop

**Affiliations:** Department of Physiology, Radboud Institute for Molecular Life Sciences (RIMLS), Radboud University Medical Center (Radboudumc), P.O. Box 9101, 6500 HB, Nijmegen, The Netherlands

**Keywords:** Cell signalling, Ion channel signalling, Nutrient signalling, Cell biology, Molecular biology, Metabolism

## Abstract

Digestion of dietary fibers by gut bacteria has been shown to stimulate intestinal mineral absorption [e.g., calcium (Ca^2+^) and magnesium (Mg^2+^)]. Although it has been suggested that local pH and short-chain fatty acid (SCFA) concentrations determine divalent cation absorption, the exact molecular mechanisms are still unknown. Therefore, this study aimed to determine the effects of SCFAs on intestinal Mg^2+^ absorption. We show that the butyrate concentration in the colon negatively correlates with serum Mg^2+^ levels in wildtype mice. Moreover, Na-butyrate significantly inhibited Mg^2+^ uptake in Caco-2 cells, while Ca^2+^ uptake was unaffected. Although Na-butyrate significantly lowered total ATP production rate, and resulted in increased phosphorylation of AMP-activated protein kinase (AMPK), inhibition of Mg^2+^ uptake by butyrate preceded these consequences. Importantly, electrophysiological examinations demonstrated that intracellular butyrate directly reduced the activity of the heteromeric Mg^2+^ channel complex, transient receptor potential melastatin (TRPM)6/7. Blocking cellular butyrate uptake prevented its inhibitory effect on Mg^2+^ uptake, demonstrating that butyrate acts intracellularly. Our work identified butyrate as novel regulator of intestinal Mg^2+^ uptake that works independently from metabolic regulation. This finding further highlights the role of microbial fermentation in the regulation of mineral absorption.

## Introduction

The absorption of magnesium (Mg^2+^) in the intestines is facilitated by different absorption pathways^1^. Passive, paracellular transport occurs in the small intestine, whereas active transport in the colon is facilitated by channels of the transient receptor potential melastatin (TRPM) cation channel family, TRPM6 and TRPM7, on the apical side^2,3^, and cyclin M4 (CNNM4) on the basolateral side of enterocytes^4^. So far, little is known about factors regulating intestinal Mg^2+^ absorption.

Oral Mg^2+^ supplementation is often insufficient to restore blood Mg^2+^ levels in diseases with increased prevalence of Mg^2+^ deficiency such as type 2 diabetes mellitus (T2DM), hypertension, cardiovascular disease and proton pump inhibitor (PPI)-induced hypomagnesemia^5–7^. Hence, there is an urgent need for alternative treatment options that stimulate intestinal Mg^2+^ absorption. Targeting the gut microbiota using dietary fibers is a promising strategy, as the fermentation of dietary fibers by gut bacteria was shown to improve intestinal Mg^2+^ absorption^8,9^. However, the underlying molecular mechanisms are still unknown.

One possible mechanism is that short-chain fatty acids (SCFAs; acetate, propionate and butyrate), derived from bacterial fermentation of complex dietary carbohydrates, are involved in the increased Mg^2+^ absorption^10^. There is increasing amount of evidence that SCFAs act as key signaling molecules between the gut microbiota and the host^10–12^. Indeed, SCFAs strengthen the mucosal barrier integrity, modulate immune cell responses and affect cholesterol, lipid and glucose metabolism in various tissues^12^. In the context of intestinal physiology, butyrate has been studied most intensively for its role as primary energy source for colonocytes and its protective effects against inflammation and colorectal cancer^13–15^.

Prebiotic fibers (e.g., oligofructose, inulin) increase the production of SCFAs and lower the intraluminal pH of the colon. These factors were shown to be beneficial for the solubility and absorption of Mg^2+^ in different experimental models^8,16,17^. As such, the gut microbiota-stimulatory effects on Mg^2+^ absorption are hypothesized to be dependent on SCFAs, either by acidification of the intestinal lumen or by affecting active transport mechanisms. Although these concepts have been widely adopted in current scientific literature, the direct effects of SCFAs on intestinal Mg^2+^ absorption have never been clarified.

This study investigated the physiological role of SCFAs on intestinal Mg^2+^ absorption by elucidating the molecular mechanisms by which SCFAs modulate Mg^2+^ uptake in the human colon cell line Caco-2, and by correlating serum Mg^2+^ levels to colonic SCFA concentrations in wildtype C57BL/6J mice.

## Results

### Colonic butyrate concentrations negatively correlate with serum Mg^2+^ levels in mice

**Figure 1:**
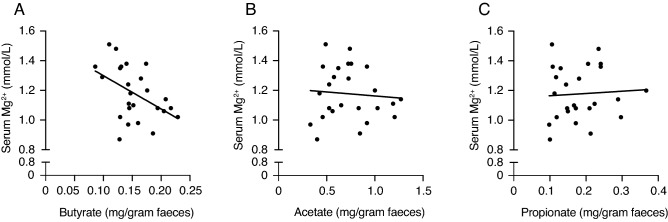
Butyrate concentrations in the colon negatively correlate with serum Mg^2+^ levels in mice. (**A–C**) Simple linear regression analyses were performed on colonic SCFAs concentrations, obtained from a previously published dataset^18^, and serum Mg^2+^ levels of 25 wildtype male C57BL/6J mice. Simple linear regression analysis between colonic concentrations of butyrate (y = −2.24x + 1.52, r^2^ = 0.22, *P* < 0.05) (**A**), acetate (y = −0.05x + 1.22, r^2^ = 0.006) (**B**), propionate (y = 0.15x + 1.15, r^2^ = 0.003) (**C**), and serum Mg^2+^ levels. Data is plotted as individual data points with the linear line indicating the relationship. *P* < 0.05 is considered significant.

To assess whether microbiota-derived SCFAs influence Mg^2+^ homeostasis, correlation analyses were performed between colonic SCFAs concentrations and serum Mg^2+^ levels in wildtype C57BL/6J mice (Fig. 1). We measured the relative concentration of SCFAs in colon samples that were collected during a previous study^18^. Linear regression analysis showed a significant inverse correlation of colonic butyrate concentrations with serum Mg^2+^ levels (*P* < 0.05,* r*^2^ = 0.22) (Fig. 1A). No correlations were found between serum Mg^2+^ levels and colonic acetate or propionate concentrations (Fig. 1B,C). No correlations were found between colonic SCFAs concentrations and serum Ca^2+^ levels (Supplementary Fig. S1).

### Butyrate treatment results in reduced ^25^Mg^2+^ uptake in Caco-2 cells

**Figure 2:**
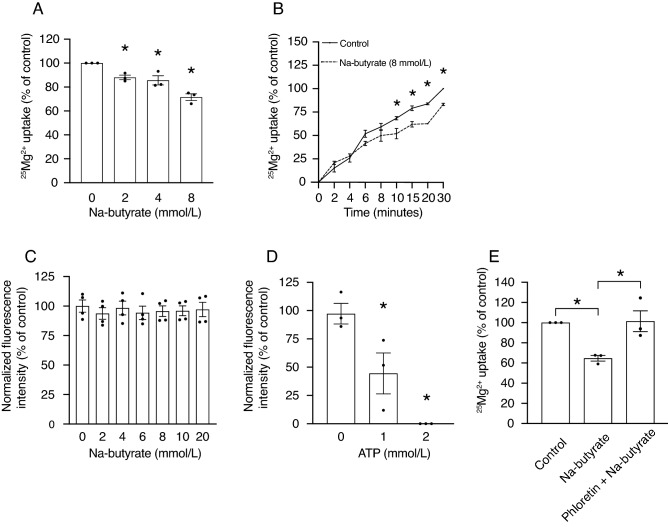
Butyrate treatment results in reduced ^25^Mg^2+^ uptake in Caco-2 cells. (**A**) ^25^Mg^2+^ uptake by Caco-2 cells after 20 min of treatment with increasing concentrations of Na-butyrate (2–8 mmol/L). Data represent mean ± SEM (n = 3, each experiment consisted of triplicates). *P < 0.05 is considered statistically significant compared to control cells using One-Way ANOVA with Dunnett’s correction for multiple testing. (**B**) Time course of ^25^Mg^2+^ uptake by Caco-2 cells treated with 8 mmol/L Na-butyrate (dashed line) or control (solid line) for 30 min. Data represent mean ± SEM (n = 3, each experiment consisted of triplicates). *P < *0.05* is considered statistically significant compared to control cells at the same timepoint using Student’s t-test. (**C**, **D**) Fluorescence intensity of 2 µmol/L magnesium green in a 1 mmol/L MgCl_2_ buffer with increasing concentrations of Na-butyrate (0–20 mmol/L) (**C**), and ATP (0–2 mmol/L) (**D**). Data represent mean ± SEM (n = 4 (**C**) and n = 3 (**D**), each experiment consisted of duplicates). (**E**) ^25^Mg^2+^ uptake by Caco-2 cells after 20 min of treatment with 8 mmol/L Na-butyrate, or 8 mmol/L Na-butyrate and 1 mmol/L phloretin. Data represent mean ± SEM (n = 3, each experiment consisted of triplicates). *P < 0.05 is considered statistically significant compared to control cells using One-Way ANOVA with Sidak’s correction for multiple testing.

To explain the underlying mechanism of the inverse correlation between colonic butyrate concentrations and serum Mg^2+^ levels, we investigated whether butyrate affects Mg^2+^ uptake in the human colon cell line Caco-2 using the heavy ^25^Mg isotope. Cells incubated for 20 min with 2, 4, or 8 mmol/L Na-butyrate had a significantly reduced ^25^Mg^2+^ uptake compared to control (100% vs. 88% ± 2 vs. 86% ± 4 vs. 72% ± 3, respectively, *P* < 0.05) (Fig. 2A). Of note, a dose–response effect was also observed within the physiological concentration range (8–40 mmol/L) (Supplementary Fig. S2)^15^. A similar reduction of ^25^Mg^2+^ uptake was observed in cells incubated with Na-propionate and Na-acetate (Supplementary Fig. S2). Further experiments were performed using the low-physiological concentration of 8 mmol/L. The inhibitory effect of Na-butyrate was time-dependent, as 8 mmol/L Na-butyrate resulted in a significantly lowered ^25^Mg^2+^ uptake between 10- and 30 min compared to control-treated cells (*P* < 0.05) (Fig. 2B). ^45^Ca^2+^ uptake was not inhibited by Na-butyrate treatment in Caco-2 cells (Supplementary Fig. S2). Next, Na-butyrate (0–20 mmol/L) was added to a magnesium green and MgCl_2_ containing buffer, in order to test if Na-butyrate would be able to bind Mg^2+^ sufficiently to decrease Mg^2+^-magnesium green binding-mediated fluorescence. None of the tested concentrations of Na-butyrate resulted in lower fluorescent intensity (Fig. 2C). As a control for our assay, addition of 1 mmol/L ATP to the magnesium green and MgCl_2_ containing buffer reduced the magnesium green fluorescence by twofold (98% ± 10 vs. 46% ± 18, *P* < 0.05) and 2 mmol/L ATP abolished the fluorescence completely (Fig. 2D). To determine whether the effects of butyrate were dependent on intracellular actions, butyrate uptake via the monocarboxylate transporter 1 (MCT1) was blocked by the known MCT1 inhibitor phloretin^19^. Similar to previous experiments, 20 min incubation with 8 mmol/L Na-butyrate significantly reduced ^25^Mg^2+^ uptake (100% vs. 65% ± 5, *P* < 0.05) and this effect was abrogated by addition of 1 mmol/L phloretin (65% ± 5 vs. 101% ± 10, *P* < 0.05) (Fig. 2E).

### Butyrate treatment results in lower ATP production by modulating cellular metabolism

**Figure 3:**
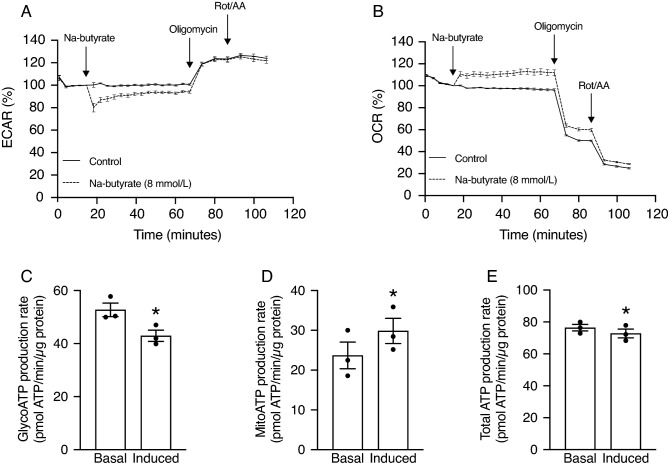
Butyrate treatment results in lower ATP production rates by modulating cellular metabolism. (**A**, **B**) Extracellular Acidification Rates (ECAR) (**A**) and Oxygen Consumption Rates (OCR) (**B**) of Caco-2 cells treated with control (solid line) or 8 mmol/L Na-butyrate (dashed line) (first arrow), followed by 2 µmol/L oligomycin (blocks OXPHOS as inhibitor of ATP synthase; second arrow) and a mixture of 0.5 µmol/L rotenone and antimycin A (blocks OXPHOS as inhibitor of mitochondrial complex I and III, respectively; third arrow), as measured with the Seahorse Xfe96 analyzer and normalized to control cells. Results of one representative experiment are shown, experiments were repeated in triplicate. Data represent mean ± SEM with 12 replicates per condition. (**C–E**) Glycolytic (**C**), mitochondrial (**D**) and total (**C**) ATP production rate of cells before (basal) and after (induced) treatment with 8 mmol/L Na-butyrate. Data depicted in (**C–E**) represent mean ± SEM, and is computed from individual traces from three independent experiments, of which representative traces of one experiment are depicted in (**A**, **B**). *P < 0.05 is considered statistically significant compared to basal using paired Student’s t-test.

Since butyrate is the primary energy source for colonocytes^20,21^, we hypothesized that butyrate treatment results in lower ^25^Mg^2+^ uptake by modulating cellular metabolism (Fig. 3). To investigate this in more detail, the effects of butyrate treatment on mitochondrial oxidative phosphorylation (OXPHOS) and glycolysis, the two main metabolic pathways responsible for ATP production, were examined using the Seahorse Xfe96 analyzer. Treatment of cells with 8 mmol/L Na-butyrate decreased the extracellular acidification rate (ECAR) (Fig. 3A) and increased oxygen consumption rate (OCR) (Fig. 3B). Quantification of the ATP production rates demonstrated that butyrate treatment resulted in a significantly decreased glycolytic ATP production (52.71 ± 2.55 vs. 42.95 ± 2.13 pmol ATP/min/µg protein, *P* < 0.05) (Fig. 3C) and increased mitochondrial ATP production (23.70 ± 3.35 vs. 29.84 ± 3.17 pmol ATP/min/µg protein, *P* < 0.05) (Fig. 3D). Together, butyrate treatment resulted in a significantly decreased total ATP production (76.41 ± 2.06 vs. 72.79 ± 2.78 pmol ATP/min/µg protein, *P* < 0.05) (Fig. 3E). In contrast, treatment of cells with either 8 mmol/L acetate or propionate did not affect total ATP production rates, but did result in decreased glycolytic and increased mitochondrial ATP production (Supplementary Fig. S3).

### Butyrate reduces ^25^Mg^2+^ uptake independent of AMPK signaling and ATP levels

In order to further understand the butyrate-induced reduction in total ATP levels, we analyzed the phosphorylation levels of AMP-activated protein kinase (AMPK). AMPK is the master energy sensor of the cell and is phosphorylated under conditions of low intracellular ATP^22^. Caco-2 cells were treated with 8 mmol/L Na-butyrate, 25 mmol/L glucose (to stimulate ATP production), or 2 µmol/L oligomycin (to inhibit ATP production) for 5, 10, and 20 min.

**Figure 4:**
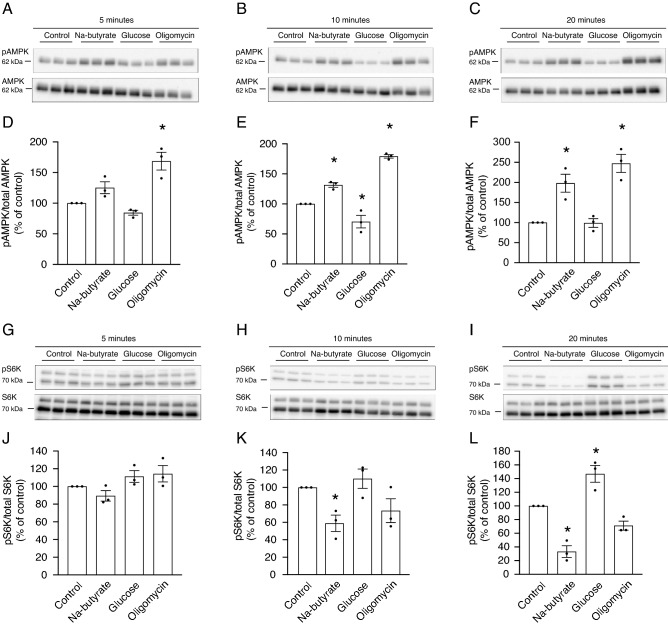
Butyrate treatment affects AMPK signaling. (**A–C**) Representative immunoblot images of pAMPK (62 kDa) and total AMPK (62 kDa) of Caco-2 cell lysates after treatment with 8 mmol/L Na-butyrate, 25 mmol/L glucose, or 2 µmol/L oligomycin for 5 min (**A**), 10 min (**B**), and 20 min (**C**). (**D–F**) Densitometry analysis of pAMPK expression corrected for total AMPK expression levels after 5 min (**D**), 10 min (**E**), and 20 min (**F**) of treatment with 8 mmol/L Na-butyrate, 25 mmol/L glucose, or 2 µmol/L oligomycin. (**G–I**) Representative immunoblot images of pS6K (70 kDa) and total S6K (70 kDa) of Caco-2 cell lysates after treatment with 8 mmol/L Na-butyrate, 25 mmol/L glucose, or 2 µmol/L oligomycin for 5 min (**G**), 10 min (**H**), and 20 min (**I**). (**J–L**) Densitometry analysis of pS6K expression corrected for total S6K expression after 5 min (**J**), 10 min (**K**), and 20 min (**L**) of treatment with 8 mmol/L Na-butyrate, 25 mmol/L glucose, or 2 µmol/L oligomycin. Data represent mean ± SEM (n = 3, each experiment consisted of triplicates). *P < 0.05 is considered statistically significant compared to control cells using One-Way ANOVA with Dunnett’s correction for multiple testing. All original immunoblots are presented in Supplementary Figs. S4–S5.

Compared to control, butyrate treatment tended to result in a 1.2 fold higher AMPK phosphorylation after 5 min (not statistically significant), while oligomycin treatment resulted in a nearly twofold higher AMPK phosphorylation (Fig. 4A,D). Yet, butyrate treatment resulted in a significantly higher AMPK phosphorylation of almost 1.5-fold after 10 min (Fig. 4B,E) and twofold after 20 min (Fig. 4C,F). Similarly, oligomycin treatment resulted in a consistently higher AMPK phosphorylation at 10 (Fig. 4B,E) and 20 min (Fig. 4C,F). Vice versa, glucose treatment, known to increase the ATP production, resulted in lower AMPK phosphorylation, which was only significantly after 10 min (Fig. 4A–F). One of the major downstream signaling pathways regulated by AMPK is the mammalian target-of-rapamycin (mTOR) pathway, involving S6K as direct downstream target^23^. As activated AMPK inhibits the mTOR pathway, the phosphorylation levels of S6K were investigated at the same timepoints. No effects of treatment with butyrate, glucose, or oligomycin, were observed on S6K phosphorylation after 5 min (Fig. 4G,J). However, butyrate treatment resulted in a significantly lower S6K phosphorylation of twofold after 10 min (Fig. 4H,K) and 2.5-fold after 20 min (Fig. 4I,L). At this latter timepoint, glucose treatment resulted in a significantly higher S6K phosphorylation with of 1.5-fold (Fig. 4I,L). All original raw western blot data is depicted in the supplementary information (Supplementary Figs. S4–S5).

**Figure 5:**
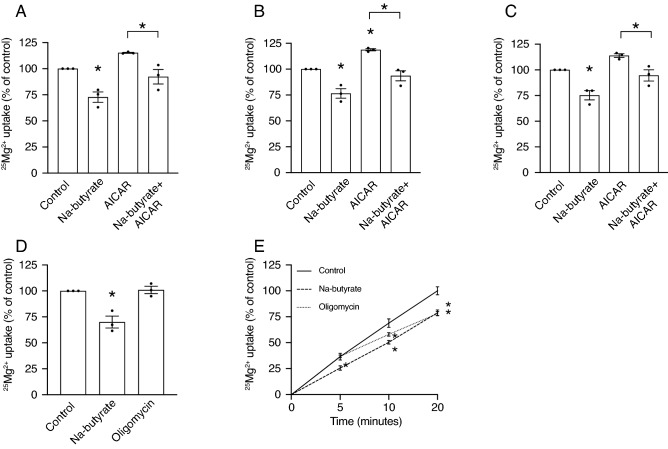
Butyrate treatment reduces ^25^Mg^2+^ uptake independent of ATP. (**A–C**) ^25^Mg^2+^ uptake by Caco-2 cells after treatment with 8 mmol/L Na-butyrate and/or 2 mmol/L AICAR for 5 min (**A**), 10 min (**B**), and 20 min (**C**). Data represent mean ± SEM (n = 3, each experiment consisted of triplicates). *P < 0.05 is considered statistically significant compared to control cells using One-Way ANOVA with Sidak’s correction for multiple testing. (**D–E**) ^25^Mg^2+^ uptake by Caco-2 cells after treatment with 8 mmol/L Na-butyrate or 2 µmol/L oligomycin after 5 min (**D**). Time course of ^25^Mg^2+^ uptake by Caco-2 cells after treatment with 8 mmol/L Na-butyrate or 2 µmol/L oligomycin at 5, 10, and 20 min (**E**). Data represent mean ± SEM (n = 3, each experiment consisted of triplicates). *P < 0.05 is considered statistically significant compared to control cells at the same timepoint using One-Way ANOVA with Dunnett’s correction for multiple testing.

To investigate whether the butyrate treatment-induced inhibition of Mg^2+^ uptake was dependent on AMPK signaling (Fig. 5), Caco-2 cells were treated for 5, 10, and 20 min with 8 mmol/L Na-butyrate and/or 2 mmol/L AICAR, a well-known AMPK-activating compound^24^. As observed in previous experiments, butyrate treatment resulted in a significantly lower ^25^Mg^2+^ uptake at all timepoints (Fig. 5A–C). However, AICAR treatment, that resulted in activation of AMPK, did not affect ^25^Mg^2+^ uptake after 5 min (Fig. 5A), and resulted in higher ^25^Mg^2+^ uptake after 10 min (Fig. 5B). Yet, combined AICAR and butyrate treatment resulted in a significantly lower ^25^Mg^2+^ uptake compared to treatment with AICAR only (Fig. 5A–C). Subsequently, we analyzed whether low intracellular ATP levels directly inhibited ^25^Mg^2+^ uptake by treating cells with oligomycin for 5, 10, and 20 min. While 5 min of butyrate treatment resulted in a significantly lower ^25^Mg^2+^ uptake of almost 1.5-fold, there were no effects of 5 min of oligomycin treatment (Fig. 5D). After 10 and 20 min, butyrate and oligomycin treatment both resulted in a significantly lower ^25^Mg^2+^ uptake (Fig. 5E).

### Intracellular butyrate directly inhibits TRPM6/7 channel activity

**Figure 6:**
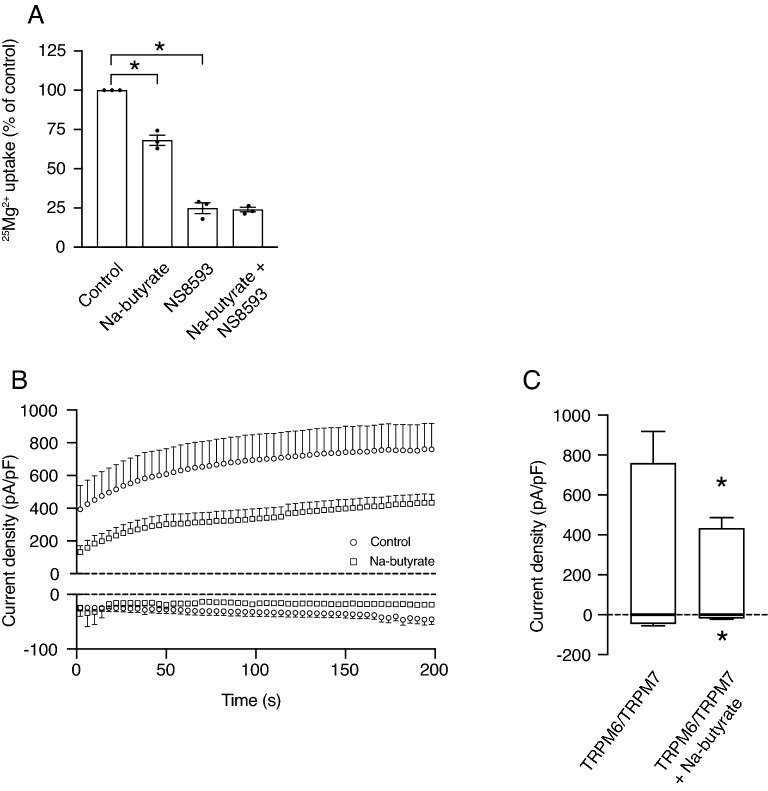
Intracellular butyrate results in lower TRPM6/7 channel activity. (**A**) ^25^Mg^2+^ uptake by Caco-2 cells after treatment with 8 mmol/L Na-butyrate and/or 30 µmol/L NS8593 after 10 min. Data represent mean ± SEM (n = 3, each experiment consisted of triplicates). * *P* < 0.05 is considered statistically significant compared to control cells using One-Way ANOVA with Sidak’s correction for multiple testing. (**B**, **C**) Whole-cell currents measured at − 80 mV and + 80 mV over time in TRPM6/7-transfected (HA-mTRPM7 pCINeo IRES GFP and HA-hTRPM6 pCINeo IRES GFP) HEK293 cells without (circles, n = 9) or with 8 mmol/L Na-butyrate (squares, n = 11) in the intracellular pipette solution (**B**). Bar graphs of current amplitudes without (Control) or with 8 mmol/L Na-butyrate (Na-butyrate) in the intracellular pipette solution (**C**). Data represent mean ± SEM. *P < 0.05 is considered statistically significant compared to control cells using Student’s t-test.

Subsequently, the potential direct effects of butyrate on Mg^2+^ uptake were investigated (Fig. 6). Over the last decade, heteromeric TRPM6/7 channels have been established as the main regulators of Mg^2+^ absorption in the colon^2,3^. In order to explore whether TRPM6/7 is involved in the butyrate treatment-inhibited Mg^2+^ uptake, Caco-2 cells were treated with 8 mmol/L Na-butyrate and the TRPM6/7 inhibitor NS8593 (30 µmol/L) for 10 minutes^3^. Importantly, treatment of Caco-2 cells with butyrate and NS8593 resulted in a fourfold lower ^25^Mg^2+^ uptake (100% vs. 24% ± 2, *P* < 0.05), similar to the effect of NS8593 alone. This suggests that butyrate treatment is exerting its effect via TRPM6/7 (Fig. 6A). Therefore, we investigated whether butyrate could directly affect TRPM6/7 channel activity. Human Embryonic Kidney (HEK293) cells transiently transfected with TRPM6/7, were subjected to whole-cell patch clamp using 8 mmol/L Na-butyrate in the patch pipette solution. Cells exposed to intracellular Na-butyrate presented significantly lower current densities over time at + 80 mV and – 80 mV, compared to control (759 ± 159 vs. 433 ± 53 pA/pF, *P* < 0.05 and − 45 ± 10 vs. − 18 ± 4 pA/pF, *P* < 0.05, respectively) (Fig. 6B,C). Of note, the effects of intracellular Na-butyrate specifically resulted in lower current densities of TRPM7, as current densities of TRPM6 were not affected (Supplementary Fig. S6). All original traces of electrophysiological recordings are depicted in the supplementary information (Supplementary Figs. S7–S9).

## Discussion

Our study demonstrates that butyrate, a short-chain fatty acid derived from bacterial fermentation, reduces intestinal TRPM6/7-mediated Mg^2+^ uptake preceding changes in metabolic regulation. This conclusion is based on the following results: (i) colonic butyrate concentrations negatively correlate with serum Mg^2+^ levels in wildtype mice; (ii) butyrate treatment results in lower ^25^Mg^2+^ uptake in the human colon cell line Caco-2; (iii) combined treatment of Caco-2 cells with butyrate and phloretin, a specific inhibitor of the butyrate transporter MCT1, prevents this lower ^25^Mg^2+^ uptake; (iv) treatment of Caco-2 cells with butyrate and NS8593, a TRPM6/7 blocker, results in a fourfold lower ^25^Mg^2+^ uptake, similar to treatment of Caco-2 cells with NS8593 alone; (v) intracellular butyrate results in significantly lower TRPM6/7 channel activity.

Fermentation of dietary carbohydrates by the gut microbiota is associated with enhanced mineral absorption^25^. In the human colon, acetate, propionate and butyrate are produced at a molar ratio of approximately 60:20:20 with concentrations ranging between 20 and 140 mmol/L^10,26^. Acetate and propionate are important modulators of lipid, glucose, and cholesterol metabolism in various tissues, whereas butyrate exerts limited systemic functions but is an important modulator of colonic homeostasis^10,15,25^. Here, we report that only colonic butyrate concentrations, but not acetate and propionate, correlated with serum Mg^2+^ levels in wildtype C57BL/6J mice. In the colon, butyrate is the primary energy source for enterocytes and acts as a histone deacetylase (HDAC) inhibitor^15^. These functions are dependent on butyrate absorption by monocarboxylate transporters (e.g., MCT1)^26,27^. We now demonstrate that butyrate results in a reduced ^25^Mg^2+^ uptake via intracellular mechanisms, as phloretin, a specific inhibitor of the butyrate transporter MCT1, abolishes the inhibitory effect of butyrate on ^25^Mg^2+^ uptake. Specifically, our results suggest that intracellular butyrate initiates a negative feedback mechanism for ^25^Mg^2+^ uptake by inhibition of TRPM6/7 channels.

We showed by whole-cell patch clamp experiments that intracellular butyrate resulted in 1.5-fold lower TRPM6/7 currents compared to untreated cells. Fatty acids have been previously reported as intracellular modulators of ion channel activity and are able to act as (anta)agonists on transient receptor potential (TRP) channels^28–31^. Poly-unsaturated fatty acids, including arachidonic acid, eicosapentaenoic acid (EPA) and docosahexaenoic acid, have been shown to inhibit TRPM8 currents in Chinese hamster ovary cells^32^. Moreover, 40 µmol/L linoleic acid (LA) significantly reduced currents at both negative and positive membrane potentials of TRPM8 and TRPV1 channels in Drosophila S2 cells^33^. Similarly, 10 µmol/L LA and EPA inhibited capsaicin-evoked currents of TRPV1 by 82% and 48% in HEK293 cells, respectively^34^. These fatty acids competitively inhibited TRPV1 currents evoked by ligands (e.g. capsaicin), suggesting a common binding site^34^. Although these studies have investigated long-chain fatty acids, our data suggest a similar mechanism for the SCFA butyrate.

Generally, two mechanisms are considered to explain effects of butyrate on TRPM6/7 activity: the SCFA may directly bind to the channel or affect the lipid bilayer^28,35^. Butyrate acts as a ligand for G-protein coupled receptors (GPCRs), such as GPR43 (FFAR2), on intestinal epithelial cells^10,13^. Activation of GPR43 by butyrate stimulates phospholipase C (PLC) activity, which in turn converts phosphatidylinositol 4,5-bisphosphate (PIP_2_) to diacylglycerol (DAG) and inositol triphosphate (IP_3_)^36^. Since TRPM7 activity is negatively regulated by hydrolysis of PIP_2_^37–39^, it could be hypothesized that butyrate indirectly inhibits TRPM7 activity by the hydrolysis of PIP_2_ via activation of GPR43 at the cell surface membrane. However, this mechanism is unlikely, since the inhibitory effect of butyrate on the TRPM6/7 channel was specific for intracellular butyrate application. The negative regulation of TRPM6/7 activity by butyrate could be caused by direct interaction with the channel, or via indirect regulation through inhibitory pathways like the PKC/RACk1 pathway^40^. Therefore, structural studies are necessary to identify if there are interaction sites of butyrate within TRPM6/7 channels.

Although we show that butyrate-mediated reduced Mg^2+^ uptake is preceding effects of butyrate treatment on metabolic regulation, we cannot exclude that reduced ATP levels contribute to the long-term inhibitory effect of butyrate treatment on ^25^Mg^2+^ uptake. Although butyrate-dependent inhibition of ^25^Mg^2+^ uptake preceded effects of butyrate treatment on ATP production, blocking ATP production by oligomycin treatment also resulted in significantly lower ^25^Mg^2+^ uptake after 10 and 20 min. At these timepoints, butyrate and oligomycin treatment both resulted in a significantly higher phosphorylation of AMPK, confirming an intracellular state of low ATP. This inhibition is similar to what is observed with AMPK activating drugs, such as metformin^41^. A 24 h pre-incubation with metformin or AICAR significantly reduced ^25^Mg^2+^ uptake in Caco-2 cells and in the renal cell line mDCT15^41^. Our results demonstrate that low intracellular ATP levels are accompanied by reduced Mg^2+^ uptake. Thus, butyrate may play a dual role in reduced Mg^2+^ uptake by affecting TRPM6/7 activity acutely, and by reducing intracellular ATP levels in a delayed response.

Our results suggest that butyrate provides a negative feedback mechanism for colonic Mg^2+^ absorption upon dietary fiber intake. Dietary fibers are known to acidify the intestinal lumen and thereby increase the solubility of Mg^2+^ in the colon^8,9,16,17,42^. When the luminal ionized Mg^2+^ concentration is higher, the permeability for Mg^2+^ in the colon should be reduced to keep Mg^2+^ absorption stable. Given that dietary fibers also increase the microbial diversity and subsequently the production of butyrate^43,44^, butyrate provides an excellent signaling molecule to reduce Mg^2+^ channel activity. Since there is oscillation of butyrate concentrations upon dietary fiber fermentation^45,46^, TRPM6/7 channels will not be constantly inhibited. Considering the fact that Mg^2+^ absorption via TRPM6/7 channels is high in the proximal colon, and this intestinal segment is dominated by butyrate-producing bacteria^10,47,48^, butyrate could act as negative regulator of Mg^2+^ absorption in this segment. Although butyrate supplementation did not result in differences in serum Mg^2+^ concentration or fecal Mg^2+^ extrusion between butyrate and control-treated mice after 1 and 14 days of treatment in a previous study^49^, our correlation analyses demonstrated that the colonic butyrate content is inversely associated with the serum Mg^2+^ levels in a physiologically relevant setting.

Our study is the first that sheds molecular insights on how SCFAs influence intestinal Mg^2+^ uptake. We show that butyrate treatment results in decreased ^25^Mg^2+^ uptake by fast inhibition of TRPM6/7 channels, preceding metabolic consequences. In particular patients with Mg^2+^ deficiency may benefit from a low molar ratio of butyrate to optimally enhance Mg^2+^ absorption.

## Materials and methods

### Animals and ethics

Serum electrolyte concentrations, and organic acid concentrations from the colonic content (fermentation broth) were determined in wildtype male C57BL/6J mice described in detail by Gommers et al.^18^. This study was approved by the animal ethics board of the Radboud University Nijmegen (RU DEC 2015-0112) and by the Dutch Central Commission for Animal Experiments (CCD, AVD103002016382). This study was performed in accordance with relevant guidelines and regulations. This study was performed in compliance with the ARRIVE guidelines.

### Colonic organic acid analysis

The organic acids concentrations were determined in the colonic content (fermentation broth) of mice. In short, the colonic content was diluted four times with 1 mol/L perchloric acid (HClO_4_, Sigma-Aldrich, USA) to release the organic acids. Proteins and fat in the fermentation broth were precipitated by ultrasonication and removed by centrifugation for 10 min at 20,000×*g*. Organic acids, including the SCFAs, were determined by high performance anion exchange chromatography (HPAEC) with UV and refractive index (RI) detection. 25 µL of the supernatant was injected on a guard column in series with two Rezex ROA Organic acids H^+^ analytical columns (Phenomenex, USA). The organic acids were eluted isocratic with 5 mmol/L sulfuric acid (H_2_SO_4_), with a flow rate of 0.60 mL/min. The column oven was held at a temperature of 60 °C. Data analysis was done with Chromeleon software version 7.2 (Thermo Scientific). Quantitative analyses were performed by using standards of the organic acids (Sigma-Aldrich, USA).

### Serum electrolyte measurements

Serum Mg^2+^ concentrations were determined using a colometric xylidyl-IL blue assay kit, according to the manufacturer’s protocol (Roche/Hitachi, Tokyo, Japan) and measured at 600 nm on a Bio-Rad Benchmark Plus microplate spectrophotometer (BioRad, Hercules, California, USA). Serum Ca^2+^ levels were measured using the *o*-cresolphthalein complexone method^50^. Briefly, *o*-cresolphthalein color reagent (Sigma, Zwijndrecht, the Netherlands) was incubated with the samples and standards and the absorbance was measured immediately at 570 nm. Both Mg^2+^ and Ca^2+^ measurements were performed using Precinorm U (Roche, Basel, Switzerland) as control.

### Cell culture

The human carcinoma cell line Caco-2 (ATCC® HTB-37™, passage number 4–21) was maintained in Dulbecco’s Modified Eagle’s Medium with 25 mmol/L HEPES and 4.5 g/L glucose (DMEM, Lonza, Belgium), supplemented with 4 mmol/L l-Glutamine (Sigma-Aldrich, Saint Louis, USA), 5% (v/v) fetal bovine serum (FBS, HyClone, GE Healthcare Life Sciences, Illinois, USA), 1% (v/v) non-essential amino acids (Biowest, MO, USA), and 1% (v/v) Penicillin–Streptomycin (Gibco, New York, USA). Cells were maintained in a 37 °C incubator with 5% (v/v) CO_2_ and were cultured for 21 days or otherwise stated. Culture medium was changed every other day and cells were passed once a week when a confluency of approximately 80% was reached. Cells were seeded at a density of 0.5 × 10^5^ cells per cm^2^. Human Embryonic Kidney cells (HEK293; ATCC®-1573™, passage number 11–18) were grown in DMEM supplemented with 10% (v/v) FBS, 10 µL/mL non-essential amino acids, and 2 mmol/L l-Glutamine.

### Magnesium green probe

The effect of Na-butyrate on Mg^2+^ binding was investigated using the cell-impermeant magnesium green pentapotassium salt (Life Technologies, Oregon, USA). 100 µmol/L stock solutions of magnesium green were prepared in MQ. The working buffer contained 2 µmol/L magnesium green dye, 115 mmol/L KCl, 20 mmol/L NaCl, 0.1 mmol/L EGTA and 10 mmol/L Tris–HCL, pH 7.2. Dye affinities for Mg^2+^ were first tested in the absence of Na-butyrate in the concentration range of 0–10 mmol/L MgCl_2_ yielding a Kd ~ 1.0 mmol/L (standard curve, data not shown). Subsequently, Na-butyrate, in the range of 0–20 mmol/L, was added to the working buffer (adjusted osmolarity with NaCl). Fluorescence was measured on a Tecan I-control Infinite200 pro (Thermo Fisher Scientific) at an absorption of 500 nm and an emission of 535 nm.

### Magnesium uptake assay

Mg^2+^ uptake by Caco-2 cells was determined using the stable ^25^Mg isotope (Cortecnet, Voisins Le Brettoneux, France). We used the non-radioactive stable isotope ^25^Mg (natural abundance 10%), which can be distinguished from ^24^Mg (natural abundance 80%) and ^26^Mg (natural abundance 10%). To this end, cells were depleted from Mg^2+^ and serum overnight. At the start of the experiment, cells were washed once with a pre-warmed basic uptake buffer (in mmol/L): 125 NaCl, 5.0 KCl, 0.5 CaCl_2_, 0.5 Na_2_HPO_4_, 0.5 Na_2_SO_4_, 15 HEPES, pH 7.5 adjusted with NaOH. Then, 500 µL of basic uptake buffer containing 1.0 mmol/L ^25^MgO was added to the cells. At each time point, cells were washed three times with ice-cold phosphate-buffered saline (PBS) and lysed in HNO_3_ (≥ 65%; Sigma-Aldrich, Steinheim, Germany) at 65 °C for 15 min. ^25^Mg^2+^ uptake by the cells was determined by inductively coupled plasma mass spectrometry (ICP-MS) analysis (Faculty of Science, Radboud University, Nijmegen). Calculations were performed based on the normalized ratio between ^25^Mg and total Mg (^24^Mg + ^25^Mg + ^26^Mg) (an increase of intracellular ^25^Mg will change the isotopic ratio compared to its normal abundance).

### Calcium uptake assay

Caco-2 cells were pre-treated with 25 µmol/L BAPTA-AM (1,2-bis(o-aminophenoxy) ethane-*N*,*N*,*N*ʹ,*N*ʹ-tetraacetic acid acetoxymethyl ester) (Invitrogen, Eugene, USA) for 30 min at 37 °C. Then, cells were washed once with warm Krebs–Henseleit bicarbonate (KHB) buffer (in mmol/L): 110 NaCl, 5 KCl, 1.2 MgCl_2_, 0.1 CaCl_2_, 10 Na-acetate, 2 NaHPO4, and 20 HEPES, pH 7.4 (NaOH), followed by 10 min incubation with KHB buffer supplemented with inhibitors of voltage-gated calcium channels, 10 μmol/L felodipine and 10 μmol/L verapamil, and 1 μCi/mL ^45^Ca (lot: 17M431A, Perkin Elmer, MA, USA) at 37 °C. Subsequently, the assay was stopped by washing the cells three times with ice-cold KHB buffer supplemented with 0.5 mmol/L CaCl_2_ and 1.5 mmol/L LaCl_3_. Cells were harvested in 0.05% (v/v) SDS solution, mixed with Opti-Flour O (Perkin Elmer, MA, USA). ^45^Ca uptake was then quantified by a liquid scintillation counter (Hidex SL 600, Hidex).

### ATP production rates

Extracellular Acidification Rates (ECAR) and Oxygen Consumption Rate (OCR) were measured using the Seahorse XFe96 Extracellular Flux analyzer (Agilent Technologies, Texas, USA). Seahorse V3 culture plates were coated with 50 µg/mL fibronectin. Subsequently, Caco-2 cells were seeded at a density of 0.5 × 10^5^ cells per cm^2^ and cultured for two days. Cells were serum starved overnight before the measurement. One hour before the run, cells were washed once with PBS, and incubated with Agilent Seahorse XF DMEM Medium pH 7.4 (103575-100; with 5 mmol/L HEPES, without phenol red and sodium pyruvate, Agilent Technologies, Texas, USA) supplemented with 10 mmol/L glucose at 37 °C, without CO_2_. After basal measurements, cells received an acute injection of 8 mmol/L Na-butyrate, Na-acetate or Na-propionate, followed by 2 μmol/L oligomycin A, and a mixture of 0.5 μmol/L rotenone and 0.5 μmol/L antimycin A. One measurement cycle consisted of 3 min incubation time and 3 min measurement time. OCR and ECAR values were normalized to total protein content. ATP production rates were calculated as described by White et al.^51^.

### Immunoblotting

Cells were lysed in ice-cold lysis buffer containing (in mmol/L): 50 Tris–HCl, 1 EGTA, 1 EDTA, 10 sodium glycerophosphate, 1 sodium orthovanadate, 10 sodium fluoride, 10 sodium pyrophosphate, 270 sucrose, and 150 NaCl with 1% (v/v) Triton X-100 and a cocktail of the following protease inhibitors: PMSF (1 mmol/L), aprotinin (1 µg/mL), leupeptin (5 µg/mL), pepstatin (1 µg/mL). Protein concentrations were determined using Bradford according to the manufacturer’s protocol (Sigma Aldrich, St. Louis, MO, USA). Samples were denaturated in laemmli buffer containing 100 mmol/L dithiothreitol (DTT) for 30 min at 37 °C, subjected to SDS-PAGE (20 µg) and transferred to polyvinylidene (PVDF) membranes. Membranes were blocked in 5% (w/v) non-fat dry milk (NFDM) dissolved in 1 × Tris-buffered saline (TBS)-T (TBS with 0.1% (v/v) Tween-20) for 45–60 min at room temperature. Subsequently, membranes were immunoblotted overnight at 4 °C with primary antibody (rabbit pAMPKα (Thr172) (1:1000, Cell Signaling Technologies, Danvers, USA, #2535), rabbit AMPKα (1:1000, Cell Signaling Technologies, Danvers, USA, #5831), rabbit pS6K(Thr389) (1:1000, Cell Signaling Technologies, Danvers, USA, #2708), rabbit S6K (1:1000, Cell Signaling Technologies, Danvers, USA, #9234). Because these antibodies have been extensively characterized and are among the most widely-used in the field, membranes were cut prior to hybridisation with antibodies to save materials. This was performed in such a manner that the edges were approximately 20 kDa from the expected size of the protein of interest. The next day, the blots were washed three times in TBS-T to remove unbound primary antibody and incubated with horseradish peroxidase (HRP) conjugated secondary antibody (PO α-rabbit 1:10,000, Sigma Aldrich, St. Louis, MO, USA) for 1 h at room temperature. After washing three times with TBS-T, the proteins were visualized with chemiluminescent reagent (SuperSignal West femto/pico, Thermo Scientific, Waltham, USA) and processed with Bio-Rad Chemidoc XRS. Optimal exposure time was determined and applied to each blot specifically to prevent under- or overexposure. Band densities were quantified with densitometric analysis using Image J software (version 2.0).

### Whole-cell patch clamp

HEK293 cells were transfected with 0.5 μg of mouse TRPM7 (HA-mTRPM7 pCINeo IRES GFP) plasmid and 0.5 μg of human TRPM6 (HA-hTRPM6 pCINeo IRES GFP) plasmid using Lipofectamine 2000 (Invitrogen) at 1:2 DNA:Lipofectamine ratio for 36–48 h. For single transfections, HEK293 cells were transfected with 1 μg of human TRPM6 (HA-hTRPM6 pCINeo IRES GFP) or 1 μg mouse TRPM7 (HA-mTRPM7 pCINeo IRES GFP) using Lipofectamine 2000 (Invitrogen) at 1:2 DNA:Lipofectamine ratio for 36–48 h. Prior to the start of the experiments, cells were seeded on glass coverslips coated with 50 μg/mL fibronectin (Roche, Mannheim, Germany). Cells were placed in the recording chamber at room temperature and selected based on the intensity of the fluorescent reporter (GFP). All experiments were undertaken and analysed using an EPC-10 amplifier and the Patchmaster software (HEKA ElectroniK GmbH, Lambrecht, Germany). The sampling interval was set to 200 ms and data was low-pass filtered at 2.9 kHz. Patch clamp pipettes were pulled from thin-walled borosilicate glass (Harvard Apparatus, March-Hugstetten, Germany) and had resistance between 1 and 3 MΩ when filled with the pipette solution containing 150 mmol/L NaCl, 10 mmol/L Na_2_EDTA, and 10 mmol/L HEPES, pH 7.3 adjusted with NaOH. The extracellular solution contained 150 mmol/L NaCl, 10 mmol/L HEPES and 1 mmol/L CaCl_2_, pH 7.4 adjusted with NaOH. 400 mmol/L stock solutions of Na-butyrate were prepared in MilliQ (MQ), with a final concentration of 8 mmol/L Na-butyrate in the pipette solution. Series resistance compensation was set to 75–95% in all experiments. From a holding potential of 0 mV, a linear 500 ms voltage ramp from − 100 to + 100 mV was applied every 2 s. Current densities were obtained by normalizing the current amplitude to the cell capacitance using Igor Pro software (Wavemetrics, Oregon, USA). Bar graphs show the current densities obtained at + 80 and − 80 mV 200 s after break in to the cell.

### Statistical analysis

Results are presented as mean ± standard error of the mean (SEM). Unless otherwise indicated, comparisons between two groups were analyzed by unpaired Student’s t-test, after testing for normal distribution using the Shapiro–Wilk test. Due to the repeated measurement-nature of the Seahorse analyzer, these were specifically tested with a paired Student’s t-test. Comparisons of more than two groups were analyzed by One-Way ANOVA followed by Dunnett’s or Sidak’s post-hoc test to correct for multiple comparisons. Correlations analyses were performed using simple linear regression. GraphPad Prism (version 8) was used to perform the statistical analyses.

## Supplementary Information

Supplementary Figures.

## Data Availability

The raw data that support the findings of this study are available from the corresponding author upon reasonable request.
