# Supplementary material for: Butyrate reduces cellular magnesium absorption independently of metabolic regulation in Caco-2 human colon cells
Source: Sci Rep. 2022 Nov 3;12:18551. doi: 10.1038/s41598-022-21683-6 (PMC9633768; doi:10.1038/s41598-022-21683-6)
Supplement: Supplementary file 1 — Supplementary Figures. [file 41598_2022_21683_MOESM1_ESM.pdf]

Supplementary Information

Butyrate reduces cellular magnesium absorption independently of metabolic regulation in Caco-2 human colon cells

Lisanne M.M. Gommers[1], Pieter A. Leermakers[1], Jenny van der Wijst[1], Sara R. Roig[1], Anastasia Adella[1], Melissa A.E. van de Wal[1], René J.M. Bindels[1], Jeroen H.F. de Baaij[1#], Joost G.J. Hoenderop[1#*]

[#] These authors contributed equally: Jeroen H.F. de Baaij and Joost G.J. Hoenderop

[1] Department of Physiology, Radboud Institute for Molecular Life Sciences (RIMLS), Radboud university medical center (Radboudumc), Nijmegen, the Netherlands.

***Corresponding author:**

Joost G.J. Hoenderop, PhD

Department of Physiology (286), Radboud university medical center

P.O. Box 9101, 6500 HB Nijmegen, The Netherlands

Email: joost.hoenderop@radboudumc.nl

Telephone: +31 (0)24 36 10580

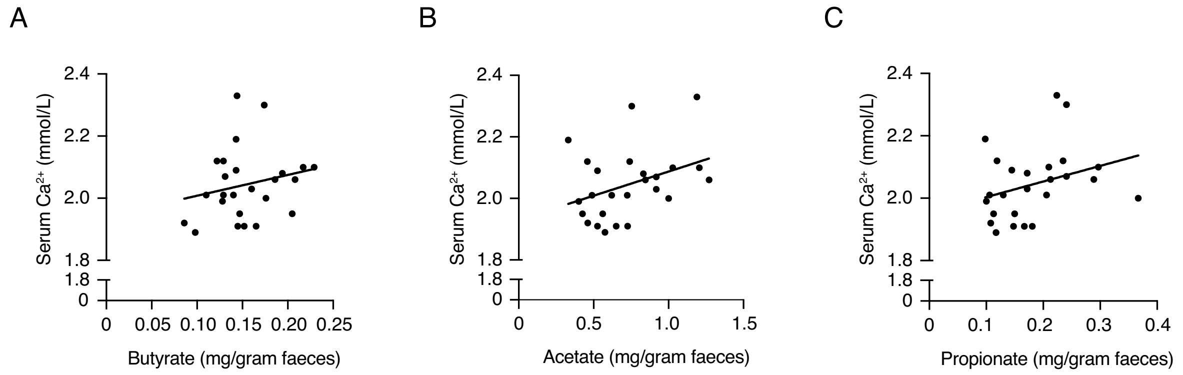

Supplementary Figure S1. Colonic SCFAs concentrations do not correlate with serum Ca^{2+} concentrations. (**A-C**) Correlation analyses were performed on colonic SCFAs concentrations, as presented in a previously published dataset (16), and serum Ca^{2+} levels of 25 wildtype male C57BL/6J mice. Simple linear regression analysis between colonic concentrations of butyrate ($y=0.67x + 1.94$, $r^2= 0.05$) (**A**), acetate ($y=0.16x + 1.93$, $r^2 = 0.14$) (**B**), propionate ($y=0.50x + 1.96$, $r^2=0.10$) (**C**), and serum Ca^{2+} levels.

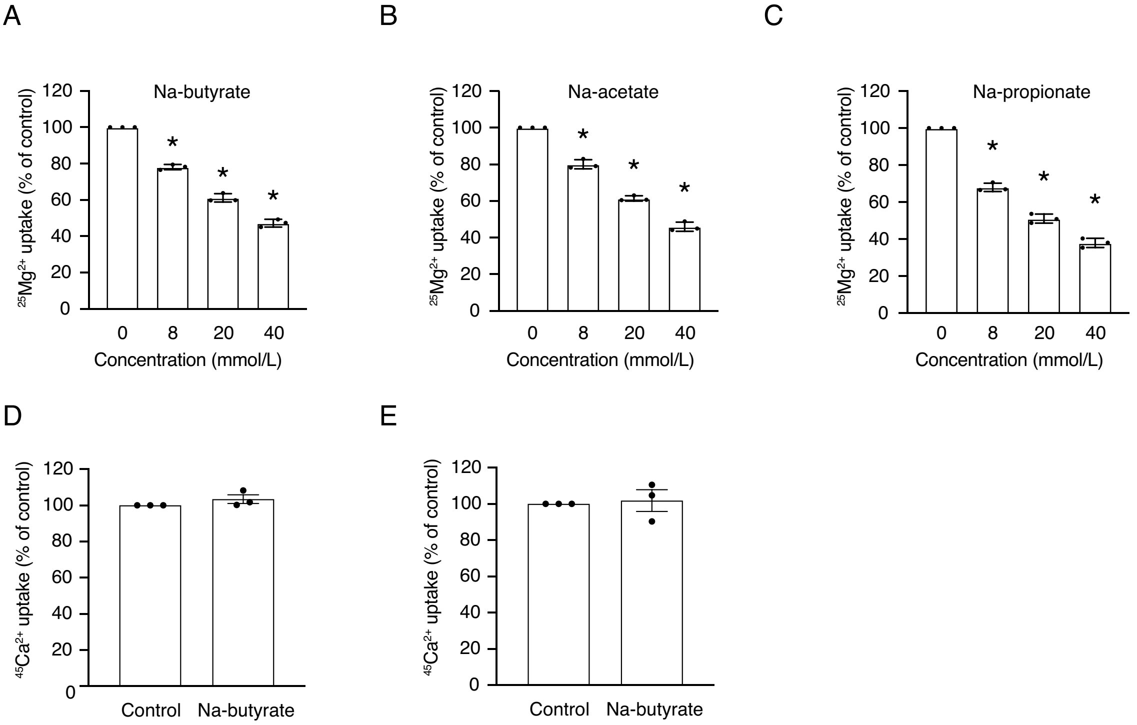

Supplementary Figure S2. SCFA treatment results in lower $^{25}Mg^{2+}$ uptake, and butyrate treatment does not result in altered $^{45}Ca^{2+}$ uptake. $^{25}Mg^{2+}$ uptake by Caco-2 cells after 20 minutes of treatment with physiological concentrations (8-40 mmol/L) of Na-butyrate (**A**), Na-acetate (**B**) and Na-propionate (**C**). Data represent mean ± SEM (n=3, each experiment consisted of triplicates). * $p < 0.05$ is considered statistically significant compared to control

cells using One-Way ANOVA with Dunnett's correction for multiple testing. (**D-E**) $^{45}Ca^{2+}$ uptake by Caco-2 cells after treatment with 8 mmol/L Na-butyrate for 6 minutes (**D**) or 36 minutes (**E**). Data represent mean ± SEM (n=3, each experiment consisted of triplicates).

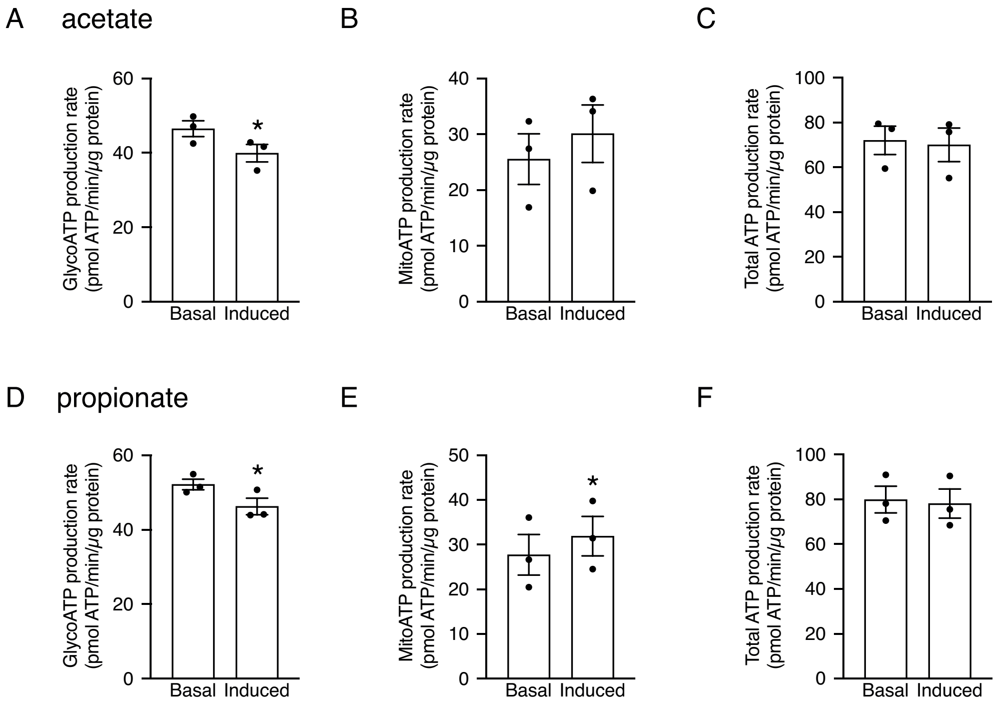

Supplementary Figure S3. Acetate and propionate treatment do not affect ATP production rates. Glycolytic- (**A, D**), mitochondrial- (**B, E**) and total ATP production rates (**C, F**) of cells before (basal) and after (induced) treatment with 8 mmol/L Na-acetate (**A-C**) or 8 mmol/L Na-propionate (**D-F**). Data represent mean ± SEM (n=3, each experiment consisted of 12 replicates). * $P < 0.05$ is considered statistically significant compared to basal using paired Student's t-test.

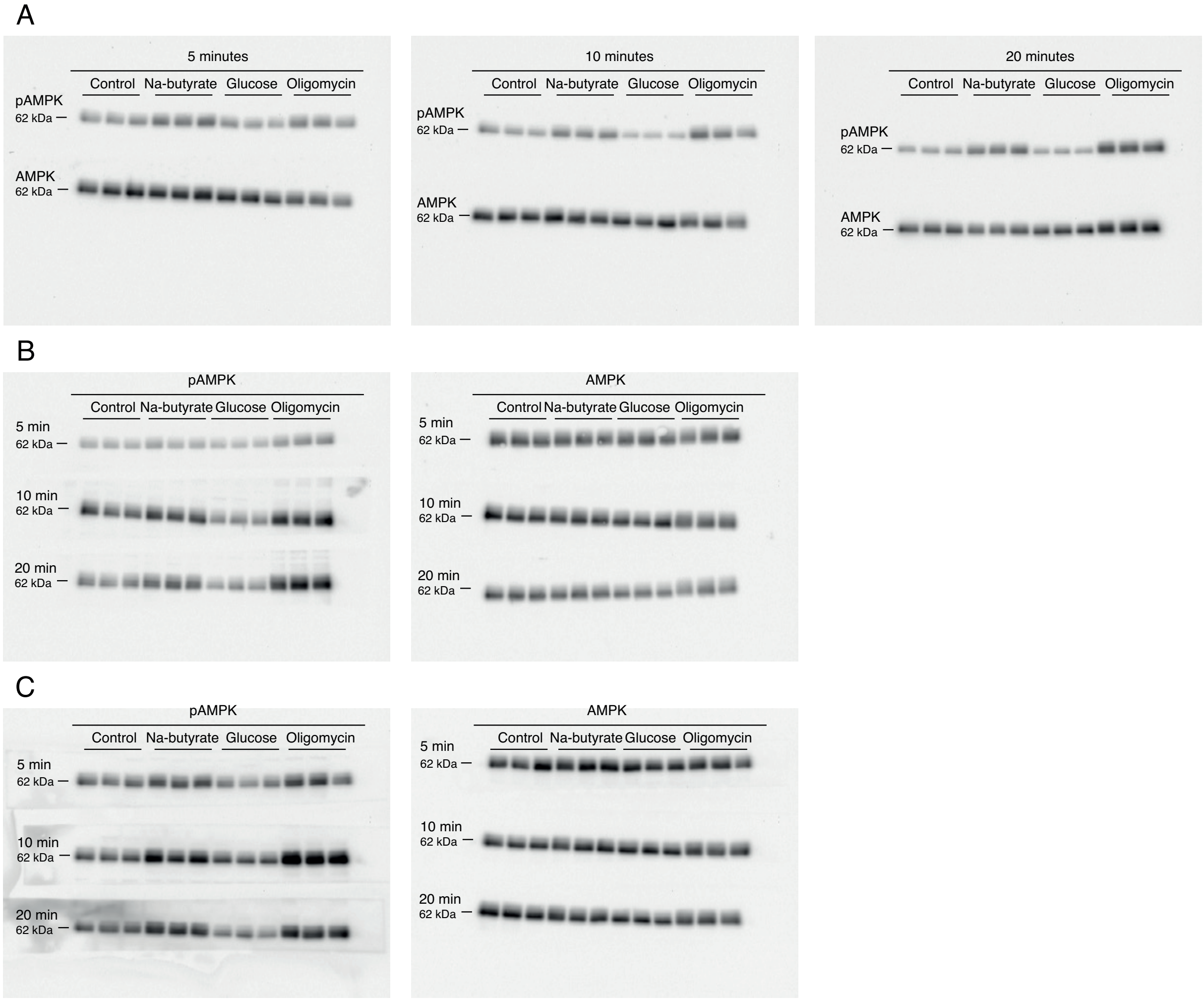

Supplementary Figure S4. Original raw western blot images pAMPK and AMPK data. Raw immunoblot images of pAMPK (62 kDa) and total AMPK (62 kDa) after treatment with 8 mmol/L Na-butyrate, 25 mmol/L glucose, or 2 µmol/L oligomycin for 5, 10, and 20 minutes. Experiments consisted of triplicates and was repeated 3 independent times; n=1 (**A**), n=2 (**B**), and n=3 (**C**).

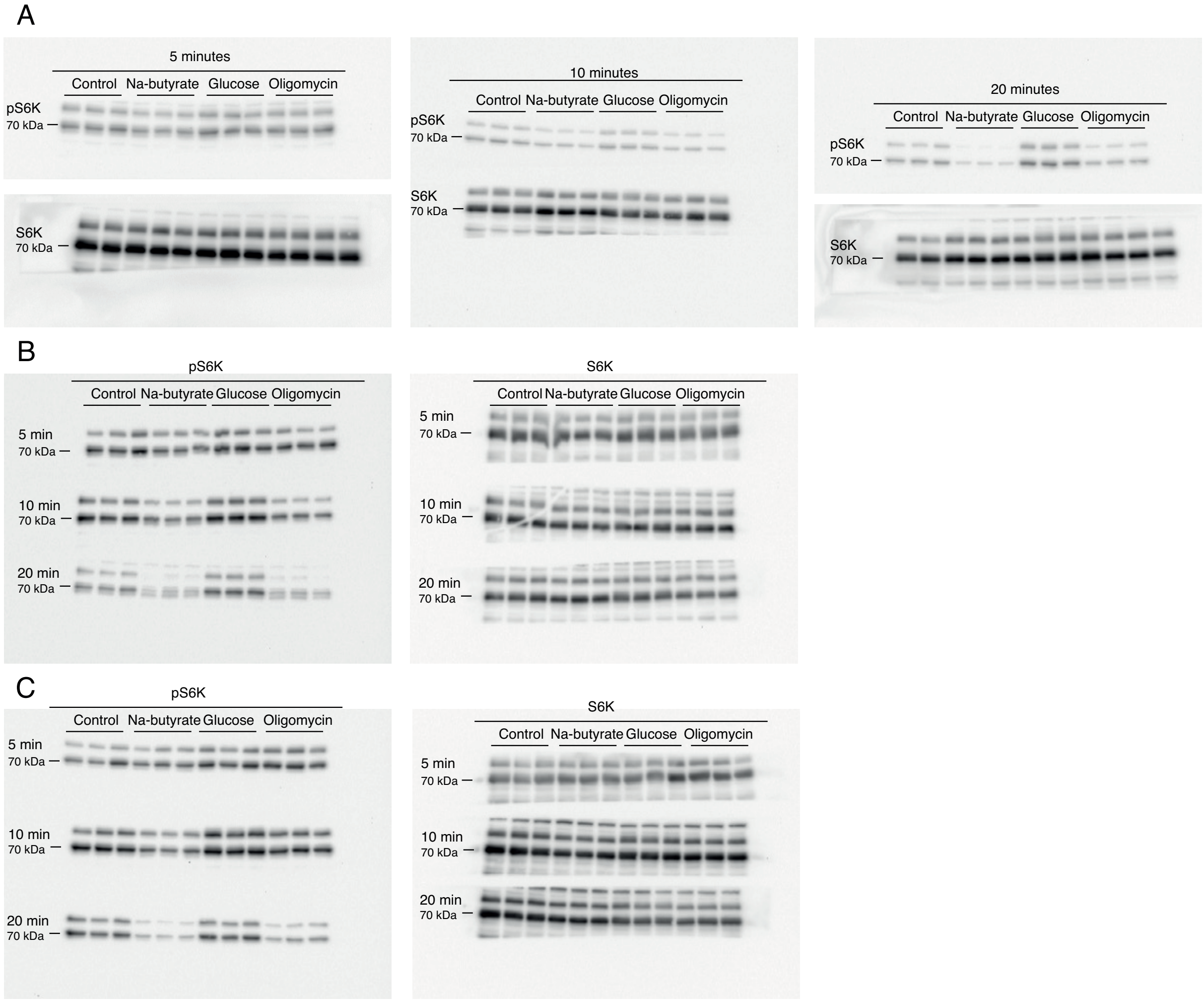

Supplementary Figure S5. Original raw western blot images pS6K and S6K data. Raw immunoblot images of pS6K (70 kDa) and total S6K (70 kDa) after treatment with 8 mmol/L Na-butyrate, 25 mmol/L glucose, or 2 µmol/L oligomycin for 5, 10, and 20 minutes. Experiments consisted of triplicates and was repeated 3 independent times; n=1 (**A**), n=2 (**B**), and n=3 (**C**).

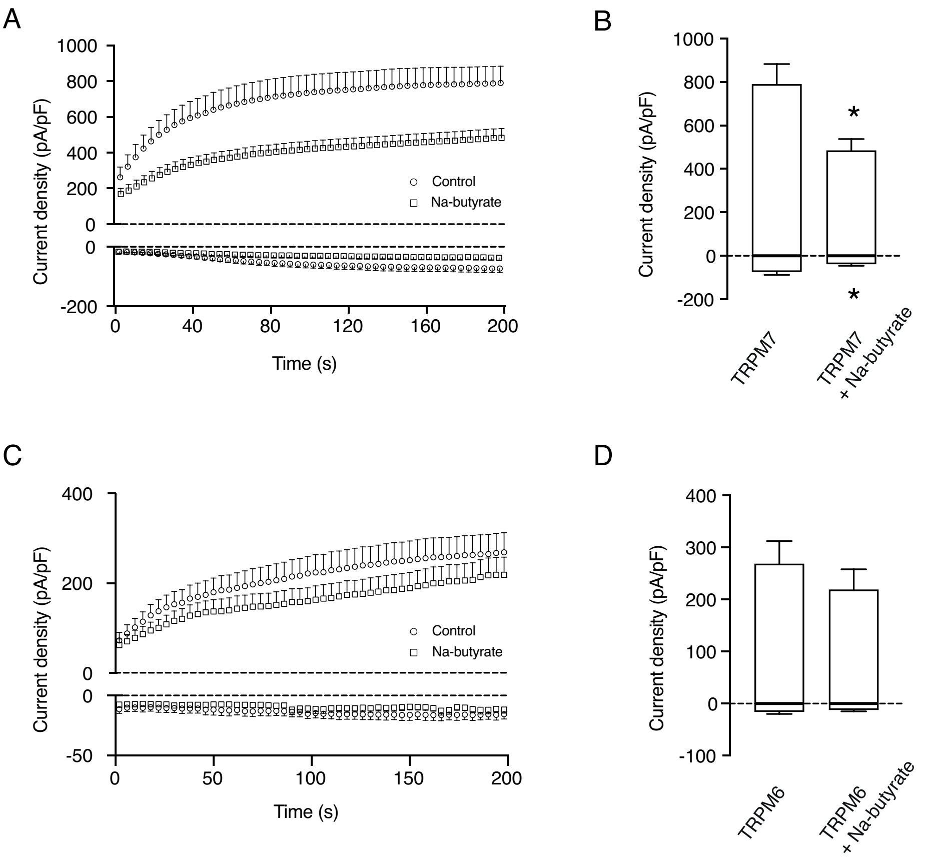

Supplementary Figure S6. Intracellular butyrate results in lower TRPM7, but not TRPM6, channel activity. (**A-B**) Whole-cell currents measured at -80 mV and +80 mV over time in TRPM7-transfected (HA-mTRPM7 pCINeo IRES GFP) HEK293 cells without (circles, n=13) or with 8 mmol/L Na-butyrate (squares, n=13) in the intracellular pipette solution (**A**). Bar graphs of current amplitudes without (Control) or with 8 mmol/L Na-butyrate (Na-butyrate) in the intracellular pipette solution (**B**). (**C-D**) Whole-cell currents measured at -80 mV and +80 mV over time in TRPM6-transfected (HA-hTRPM6 pCINeo IRES GFP) HEK293 cells without (circles, n=14) or with 8 mmol/L Na-butyrate (squares, n=12) in the intracellular pipette solution (**C**). Bar graphs of current amplitudes without (Control) or with 8 mmol/L Na-butyrate (Na-butyrate) in the intracellular pipette solution (**D**). Data represent mean ± SEM. * $P < 0.05$ is considered statistically significant compared to control cells using Student's t-test.

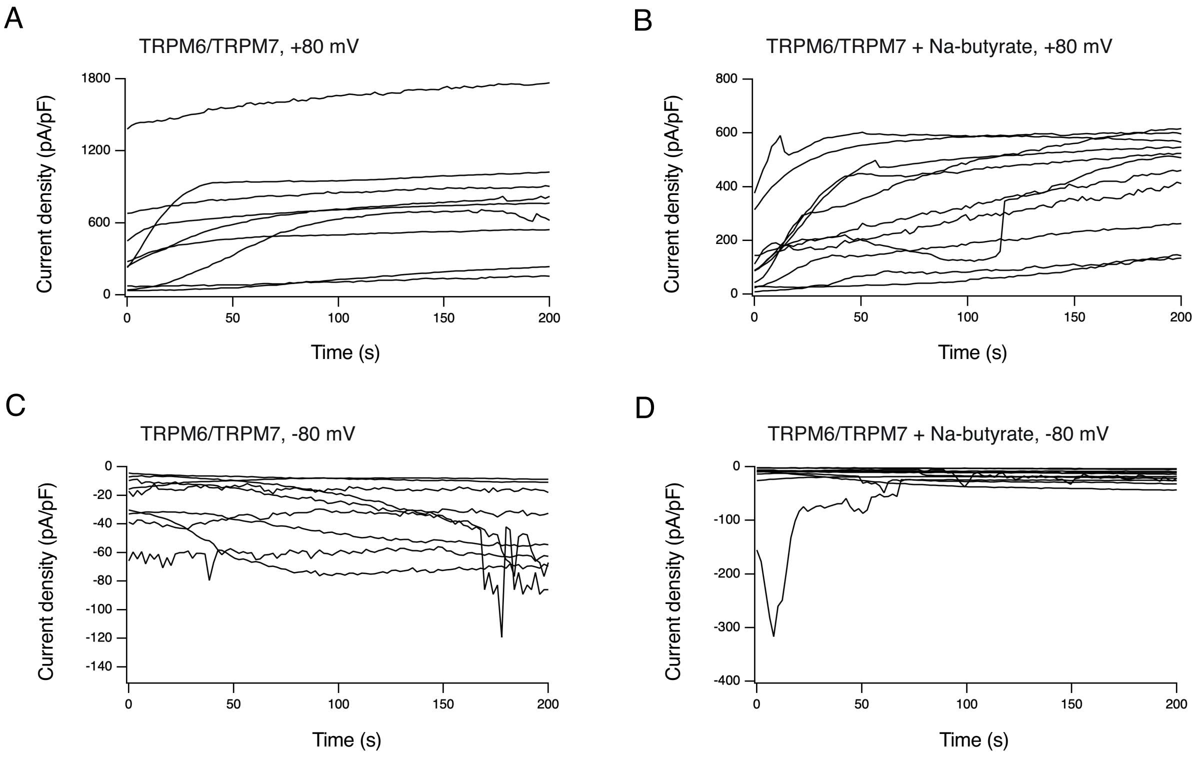

Supplementary Figure S7. Original traces of electrophysiological recordings of TRPM6/7-transfected HEK293 cells. Original traces of electrophysiological recordings measured in TRPM6/7-transfected (HA-mTRPM7 pCINeo IRES GFP and HA-hTRPM6 pCINeo IRES GFP) HEK293 cells at +80 mV over time without (**A**) or with 8 mmol/L Na-butyrate (**B**) in the intracellular pipette solution. Original traces of electrophysiological recordings measured in TRPM6/7-transfected HEK293 cells at -80 mV over time without (**C**) or with 8 mmol/L Na-butyrate (**D**) in the intracellular pipette solution.

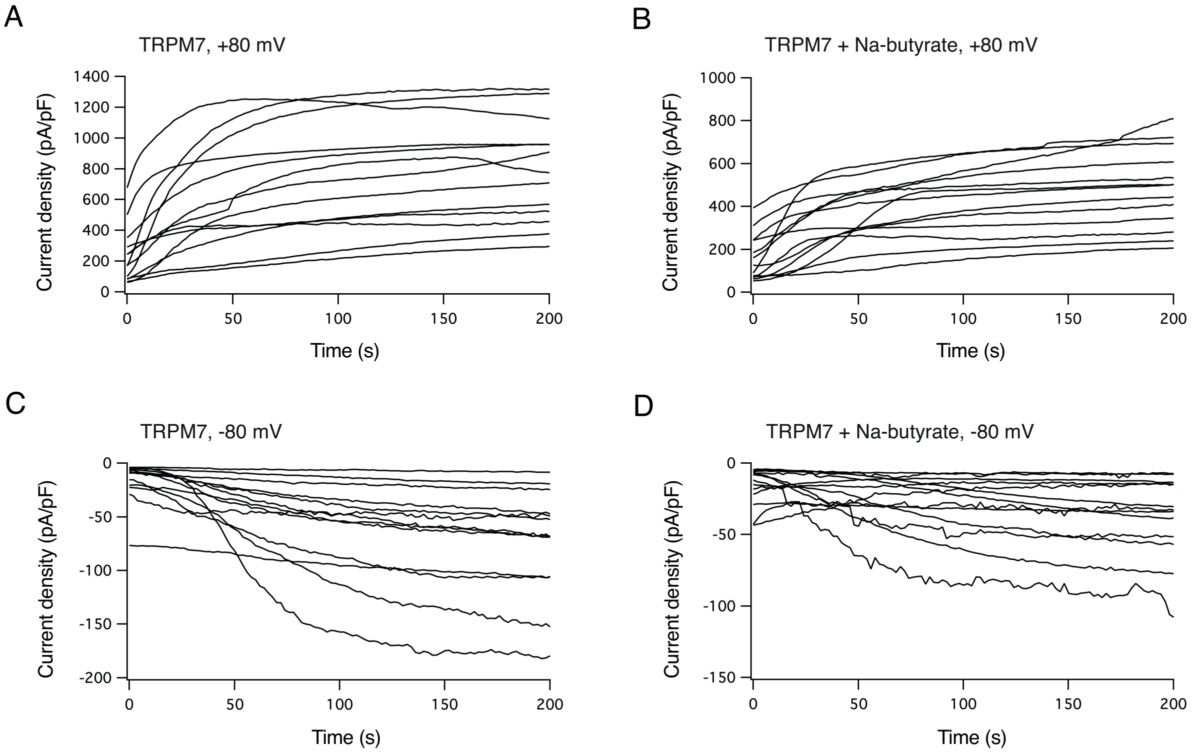

Supplementary Figure S8. Original traces of electrophysiological recordings of TRPM7-transfected HEK293 cells. Original traces of electrophysiological recordings measured in TRPM7-transfected (HA-mTRPM7 pCINeo IRES GFP) HEK293 cells at +80 mV over time without (**A**) or with 8 mmol/L Na-butyrate (**B**) in the intracellular pipette solution. Original traces of electrophysiological recordings measured in TRPM7-transfected HEK293 cells at -80 mV over time without (**C**) or with 8 mmol/L Na-butyrate (**D**) in the intracellular pipette solution.

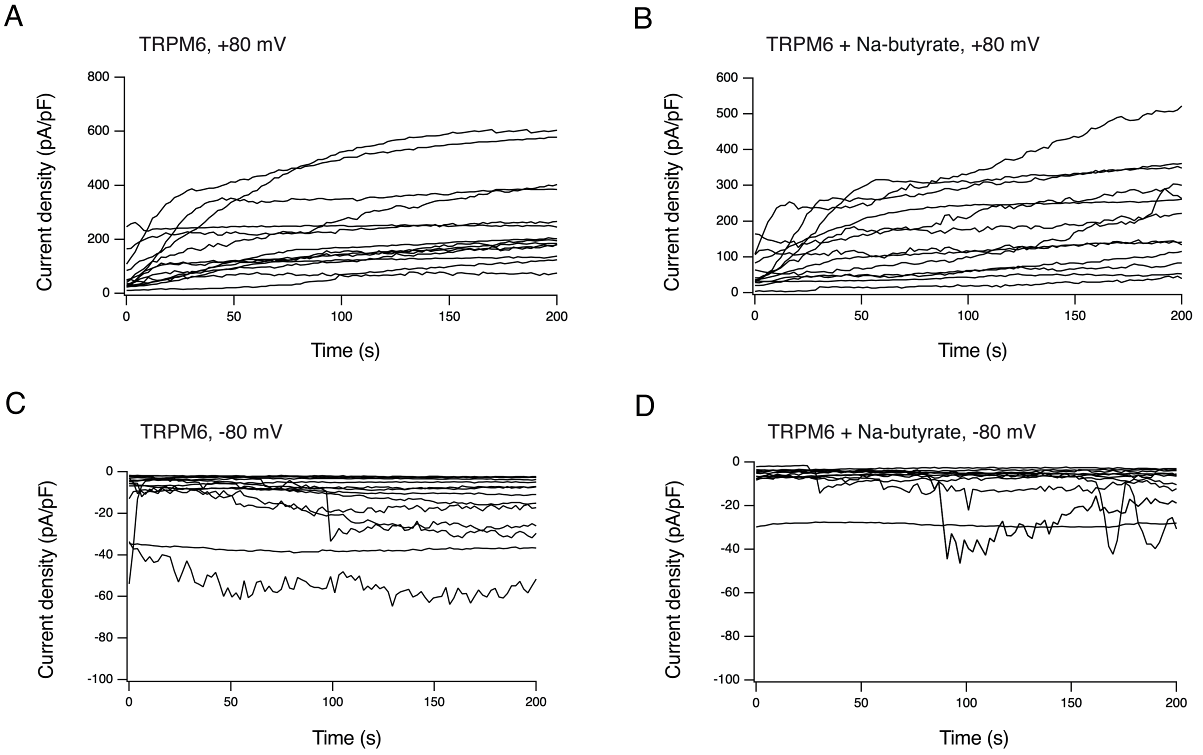

Supplementary Figure S9. Original traces of electrophysiological recordings of TRPM6-transfected HEK293 cells. Original traces of electrophysiological recordings measured in TRPM6-transfected (HA-hTRPM6 pCINeo IRES GFP) HEK293 cells at +80 mV over time without (**A**) or with 8 mmol/L Na-butyrate (**B**) in the intracellular pipette solution. Original traces of electrophysiological recordings measured in TRPM6-transfected HEK293 cells at -80 mV over time without (**C**) or with 8 mmol/L Na-butyrate (**D**) in the intracellular pipette solution.
